# Structures of five salt forms of di­sulfonated monoazo dyes

**DOI:** 10.1107/S2053229620012735

**Published:** 2020-09-23

**Authors:** Heather C. Gardner, Alan R. Kennedy, Karen M. McCarney, Edward Staunton, Heather Stewart, Simon J. Teat

**Affiliations:** a Westchem, Department of Pure & Applied Chemistry, University of Strathclyde, 295 Cathedral Street, Glasgow G1 1XL, Scotland; b Advanced Light Source, Lawrence Berkeley National Laboratory, 1 Cyclotron Road, Berkeley, CA 94720, USA

**Keywords:** dyes, salt forms, sulfonates, monoazo, coordination polymers, crystal structure

## Abstract

The structures of five *s*-block metal salt forms of three di­sulfonated monoazo dyes are presented. These are poly[di-μ-aqua-di­aqua­[μ_4_-3,3′-(di­azane-1,2-di­yl)bis­(benzene­sulfonato)]disodium(I)], [Na_2_(C_12_H_8_N_2_O_6_S_2_)(H_2_O)_4_]_*n*_, (I), *catena*-poly[[tetra­aqua­calcium(II)]-μ-3,3′-(di­azane-1,2-di­yl)bis­(benzene­sulfonato)], [Ca(C_12_H_8_N_2_O_6_S_2_)(H_2_O)_4_]_*n*_, (II), *catena*-poly[[[di­aqua­calcium(II)]-μ-2-(4-amino-3-sulfonato­phen­yl)-1-(4-sulfonato­phen­yl)diazenium] dihydrate], {[Na(C_12_H_10_N_3_O_6_S_2_)(H_2_O)_2_]·2H_2_O}_*n*_, (III), hexa­aqua­magnesium bis­[2-(4-amino-3-sulfonato­phen­yl)-1-(4-sulfonato­phen­yl)diazenium] octa­hydrate, [Mg(H_2_O)_6_](C_12_H_10_N_3_O_6_S_2_)_2_·8H_2_O, (IV), and poly[[{μ_2_-4-[2-(4-amino-2-methyl-5-meth­oxy­phen­yl)diazen-1-yl]benzene-1,3-di­sulfonato}di-μ-aqua-di­aqua­barium(II)] dihydrate], {[Ba(C_14_H_13_N_3_O_7_S_2_)(H_2_O)_4_]·2H_2_O}_*n*_, (V). Compound (III) is that obtained on crystallizing the commercial dyestuff Acid Yellow 9 [74543-21-8]. The Mg species is a solvent-separated ion-pair structure and the others are all coordination polymers with bonds from the metal atoms to sulfonate groups. Compound (I) is a three-dimensional coordination polymer, (V) is a two-dimensional coordination polymer and both (II) and (III) are one-dimensional coordination polymers. The coordination behaviour of the azo ligands and the water ligands, the dimensionality of the coordination polymers and the overall packing motifs of these five structures are contrasted to those of mono­sulfonate monoazo congers. It is found that (I) and (II) adopt similar structural types to those of mono­sulfonate species but that the other three structures do not.

## Abstract 2

The structures of five *s*-block metal salt forms of three di­sulfonated monoazo dyes are presented. The coordination behaviour of the azo ligands and the water ligands, the dimensionality of the coordination polymers and the overall packing motifs of these five structures are contrasted to those of mono­sulfonate monoazo congers. It is found that two of the com­pounds adopt similar structural types to those of mono­sulfonate species but that the other three structures do not.

## Introduction   

Azo com­pounds have a long history of use as both dyes and pigments. One of the commonest subclasses is that of sulfon­ated azo species, where the sulfonate group is typically added to aid water solubility and/or to decrease toxicity (Hunger *et al.*, 2003 ▸). Despite being widely referred to as organic colourants, the commercial products of sulfonated azo species are commonly metal com­plexes and often *s*-block metal salt forms (Christie & Mackay, 2008 ▸). Even before large-scale crystallographic studies were available, it was recognized that small structural changes systematically changed the colour and material properties of such dyestuffs (Greenwood *et al.*, 1986 ▸). These structure–property relationships led to an inter­est in more detailed structural investigations. A reasonable number of crystal structures of the salt forms of mono­sulfonated azo dyes and even pigments are now known (*e.g.* Kennedy *et al.*, 2000 ▸, 2004 ▸, 2009 ▸; Tapmeyer *et al.*, 2020 ▸; Aiken *et al.*, 2013 ▸). However, far fewer relevant structures of di­sulfonated azo species are known, despite these being commercially commonplace. The only azo­benzene-based di­sulfonate structures that we are aware of are those of azo­benzene-4,4′-di­sulfonate (Soegiarto & Ward, 2009 ▸; Soegiarto *et al.*, 2010 ▸, 2011 ▸). In these structures, the di­sulfonate ions are utilized as framework hosts for a series of functional organic guests and thus they are not of particular relevance to commercial colourant materials. Some *s*-block metal salt structures of more com­plicated di­sulfonated dyes, with naphthalene- rather than azo­benzene-based azo fragments, are also known (*e.g.* Black *et al.*, 2019 ▸; Kennedy *et al.*, 2006 ▸; Ojala *et al.*, 1994 ▸). The azo moiety in all these examples exists in the hydrazone tautomeric form and in all cases both sulfonate groups lie on only one ring system at one end of the azo bond. The only colourant relevant di­sulfonate structures with sulfonate groups on both the ring systems, at either end of an azo bond, are the Ca lake structures of Pigment Yellow 183 and Pigment Yellow 191 determined by Schmidt and co-workers (Ivashevskaya *et al.*, 2009 ▸; Schmidt *et al.*, 2009 ▸). These are relatively com­plex materials with pyrazolone groups between the two sulfonated aryl rings. Herein we present five new structures of *s*-block metal salt forms of azo­benzene di­sulfonate derivatives (Scheme 1), namely, [Na_2_ *L*1(OH_2_)_4_]_*n*_, (I), and [Ca*L*1(OH_2_)_4_]_*n*_, (II), where *L*1 is azo­benzene-3,3′-di­sulfonate; {[Na*L*2(OH_2_)_2_]·2H_2_O}_*n*_, (III),[Chem scheme1] and [Mg(OH_2_)_6_][*L*2]_2_·8H_2_O, (IV), where *L*2 is 4-amino­di­azen­iumyl­benzene-3,4′-di­sulfonate; and {[Ba*L*3(OH_2_)_4_]·2H_2_O}_*n*_ (V), where *L*3 is 4-amino-2-methyl-5-meth­oxy­azo­ben­zene-2′,4′-di­sulfonate. Structure (III) is notable as it was obtained from recrystallizing the commercial dyestuff Acid Yellow 9 [74543-21-8].
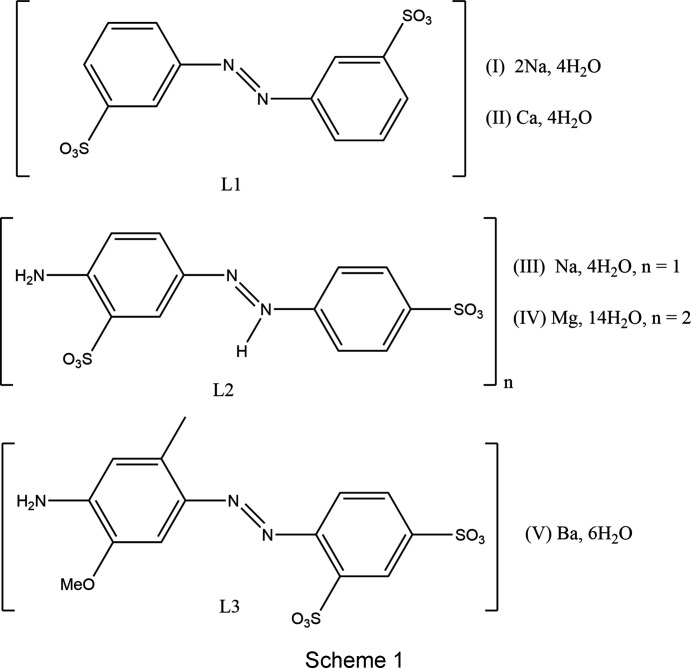


## Experimental   

### Synthesis and crystallization   

The Raman spectra of solid samples were measured using a Reinshaw Ramascope 2000 instrument with excitation at 785 nm. IR samples were prepared as KBr discs and spectra were measured using a Nicolet Avatar 360 FT–IR.

The Na salt of azo­benzene-3,3′-di­sulfonate, (I), was pro­duced by the alkaline reduction of 3-nitro­benzene­sulfonic acid by glucose (Galbraith *et al.*, 1951 ▸). Yellow crystals suitable for analysis were obtained directly from the aqueous reaction mixture. IR (KBr): 1645 (*br*), 1470, 1419, 1235, 1199, 1107, 1081, 1045, 999, 902, 810, 712, 685, 620, 569, 528 cm^−1^. Raman: 1477, 1413, 1183, 1163, 1104, 995, 283 cm^−1^. Microanalysis found (expected) (%): C 31.57 (31.44), H 3.56 (3.53), N 5.90 (6.11), S 13.66 (13.99).

The Ca salt (II) was prepared by adding excess CaCl_2_ to an aqueous solution of (I). After filtration, the resulting solution deposited yellow–orange crystals of (II) after slow evaporation (four weeks). IR (KBr): 1629, 1465, 1204, 1102, 1076, 1050, 999, 794, 712, 682, 615 cm^−1^. Raman: 1592, 1420, 1376, 1325, 1198, 1162, 1124, 978, 822, 602, 381, 350, 277 cm^−1^. The crystals were somewhat hygroscopic and an acceptable micro­analysis was not obtained.

The monosodium salt of Acid Yellow 9 was purchased from Sigma–Aldrich and recrystallized from water to give fibrous red crystals of (III). The Mg salt (IV) was prepared by adding an equimolar amount of MgCl_2_ to an aqueous solution of the monosodium salt of Acid Yellow 9. After filtering off the initial dark precipitate, allowing the remaining solution to evaporate to dryness gave red crystals of (IV). IR (KBr): 1625, 1574, 1528, 1392, 1162, 1008, 879 cm^−1^.

The free acid equivalent of (V) was provided by Dystar UK. Treatment of an aqueous solution with Ba(OH)_2_ gave an orange solution. After several attempts, a simple slow evaporation (approximately four weeks) from water gave a few suitable orange crystals of (V).

### Refinement   

Crystal data, data collection and structure refinement details are summarized in Table 1 ▸. Data for (III) were measured at the Daresbury SRS Station 9.8 (Cernik *et al.*, 1997 ▸) and for (V), data were measured by the UK National Crystallography Service (Cole & Gale, 2012 ▸).

Disorder models were used for one non-metal-bound water mol­ecule of both (III) and (IV), and also for one SO_3_ group of (IV). In all cases, a two-site model was used and site-occupancy factors were refined. Suitable restraints and constraints were applied to the bond lengths and displacement parameters of the disordered units to ensure that they displayed approximately normal behaviour.

For all structures, H atoms bound to C atoms were placed in the expected geometric positions and treated in riding mode, with C—H = 0.95 Å and *U*_iso_(H) = 1.2*U*_eq_(C) for C—H groups, and C—H = 0.98 Å and *U*_iso_(H) = 1.5*U*_eq_(C) for CH_3_ groups. H atoms bound to N or O atoms were located by difference synthesis and placed accordingly. For (III) and (IV), H atoms bound to N atoms were refined freely and isotropically. For (V), the N—H distances were restrained to 0.88 (1) Å. All water H atoms were restrained such that O—H = 0.88 (1) Å and H⋯H = 1.33 (2) Å. For the water H atoms of (V) and the H atoms of the disordered groups, *U*_iso_ values were allowed to ride on the parent O atom and for all other water H atoms, *U*_iso_ values were allowed to refine freely.

## Results and discussion   

Previous work on the salt forms of mono­sulfonated dyes and pigments has shown that many structural features can be predicted from knowledge of the cation identity and the position of the sulfonate group (Kennedy *et al.*, 2009 ▸, 2012 ▸). With respect to *L*1 and the metal cations used herein, relevant observations on mono­sulfonated species with a similar *meta* relationship between the azo and SO_3_ groups are as follows. Na structures are expected to feature high-dimensionality coordination polymers with both SO_3_ and H_2_O groups bridging between Na centres. However, if metal-to-sulfonate bonds exist at all, then Ca structures should either be nonpolymeric entities or simple one-dimensional polymers with H_2_O ligands adopting only terminal positions. *L*2 has both *meta* and *para* relationships between its azo and SO_3_ groups. Again extrapolation from what is known of mono­sulfonated azo salt forms would suggest that for *L*2 an Mg species should be a solvent-separated ion-pair structure with no Mg—O_3_S bonds, whilst Na species should have a high-dimensional coordination polymer structure similar to those predicted for an Na salt of *L*1 above (Kennedy *et al.*, 2004 ▸). In all cases, the overall packing should feature simple alternating layers of hydro­philic groups (*e.g.* cations, SO_3_ and H_2_O) and hydro­phobic groups (the aryl azo body of the anions) (Kennedy *et al.*, 2009 ▸).

**Figure 1:**
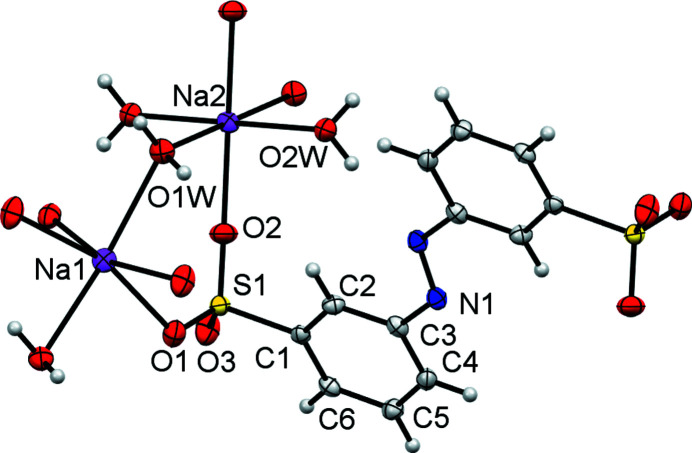
The asymmetric unit of (I) expanded to show the coordination shell about Na1 and Na2, and the conformation of *L*1. Non-H atoms are shown as 50% probability displacement ellipsoids and H atoms are drawn as small spheres of arbitrary size.

**Figure 2:**
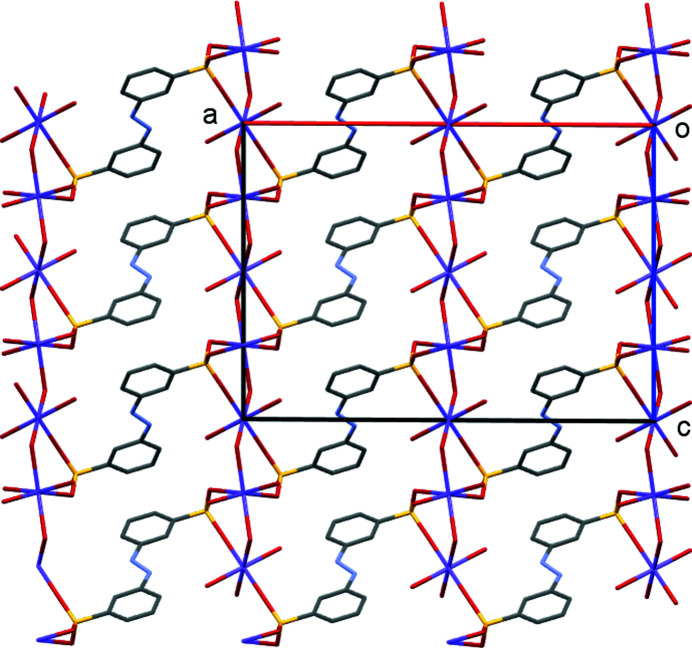
Packing diagram of (I), viewed down the *b* axis. Note the alternating hydro­phobic and hydro­philic layers that lie parallel to the *bc* plane.

The structure of di­sulfonate (I) fits well with these predictions from mono­sulfonates. It is indeed a three-dimensional coordination polymer with both SO_3_ and H_2_O groups bridging between metal centres, and it forms a simple layered structure as expected. In more detail, the asymmetric unit of (I) contains two separate Na sites, both of which occupy special positions (Na1 sits on a twofold axis and Na2 on a centre of symmetry in the space group *C*2/*c*). It also contains two water ligands and half of an *L*1 dianion. A crystallographic centre of symmetry is located at the centre of the azo bond, giving a planar dianion with mutually *anti* SO_3_ groups (Fig. 1 ▸). As can be seen from Table 2 ▸, each Na centre is approximately octa­hedral, with Na1 bonding to two bridging water mol­ecules and to four O atoms of four different *L*1 dianions. Na2 bonds to two O atoms of two *L*1 dianions and to four water ligands, two of which form terminal bonds and two of which bridge to Na1 centres. Note that the bond lengths involving Na1 are systematically longer than those of Na2 [ranges 2.4174 (19)–2.5019 (18) and 2.3340 (18)–2.4480 (17) Å for Na1 and Na2, respectively]. The SO_3_ units each form three bonds to Na centres, one from each O atom. Within the hydro­philic layers, pairs of Na1 centres are linked by eight-membered [NaOSO]_2_ rings, whilst the Na1 and Na2 centres are linked by six-membered [NaOSONaO] rings, with both bridging sulfonate and water ligands. As can be seen from Fig. 2 ▸, the layers expand parallel to the *bc* plane, with the di­sulfonate dianions bridging between neighbouring hydro­philic layers to give the overall three-dimensional coordination polymer. The hydrogen-bond details for (I) are given in Table 3 ▸.

**Figure 3:**
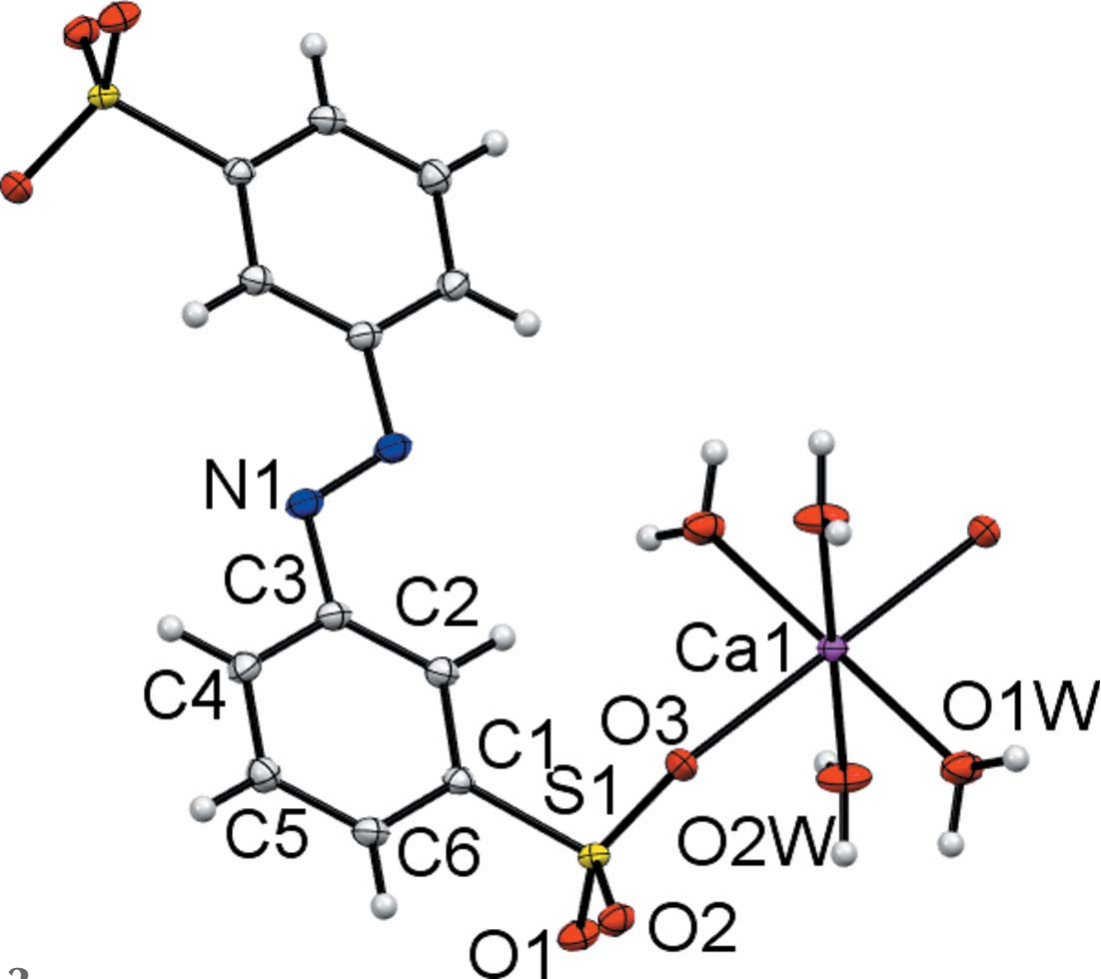
The asymmetric unit of (II) expanded to show the coordination shell about Ca1 and the conformation of *L*1. Non-H atoms are shown as 50% probability displacement ellipsoids and H atoms are drawn as small spheres of arbitrary size.

**Figure 4:**
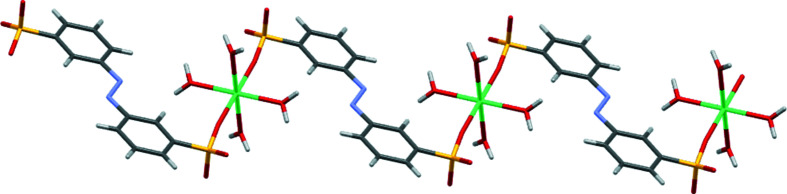
Part of the one-dimensional coordination polymer of (II).

**Figure 5:**
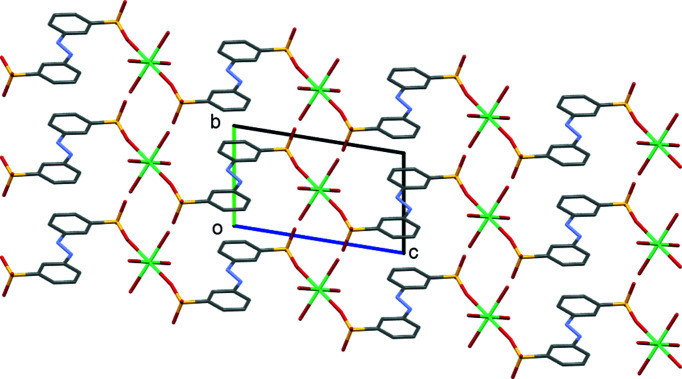
Packing diagram of (II), viewed down the *a* axis. Note the alternating hydro­phobic and hydro­philic layers that lie parallel to the *ab* plane.

The asymmetric unit of (II) contains half of an *L*1 dianion, two water ligands and a Ca site. Both the Ca1 site and the centre of the azo N=N bond occupy crystallographic inversion centres. As with (I), this gives a planar dianion with *anti* SO_3_ groups and an octa­hedral metal centre (Fig. 3 ▸ and Table 4 ▸). Ca1 forms bonds to O atoms from two *trans* SO_3_ groups and to four terminal water ligands. Each SO_3_ group makes a single Ca—O bond and thus the di­sulfonate dianion links Ca centres into a one-dimensional coordination polymer (Fig. 4 ▸). These features combine to give the layered structure shown in Fig. 5 ▸. Within the hydro­philic layers, hydrogen bonding between the water ligands and the two noncoordinating O atoms of SO_3_ link neighbouring coordination chains (Table 5 ▸). Thus, structure (II) also follows the rules proposed for mono­sulfonated azo dye salts. There are Ca—O_3_S bonds, but these are relatively few in number and, even with the two-headed nature of the di­sulfonate ligand, they combine to give only a one-dimensional coordination polymer. The H_2_O ligands take no part in bridging between metal centres and the overall packing motif is one of simple alternating hydro­phobic and hydro­philic layers.

**Figure 6:**
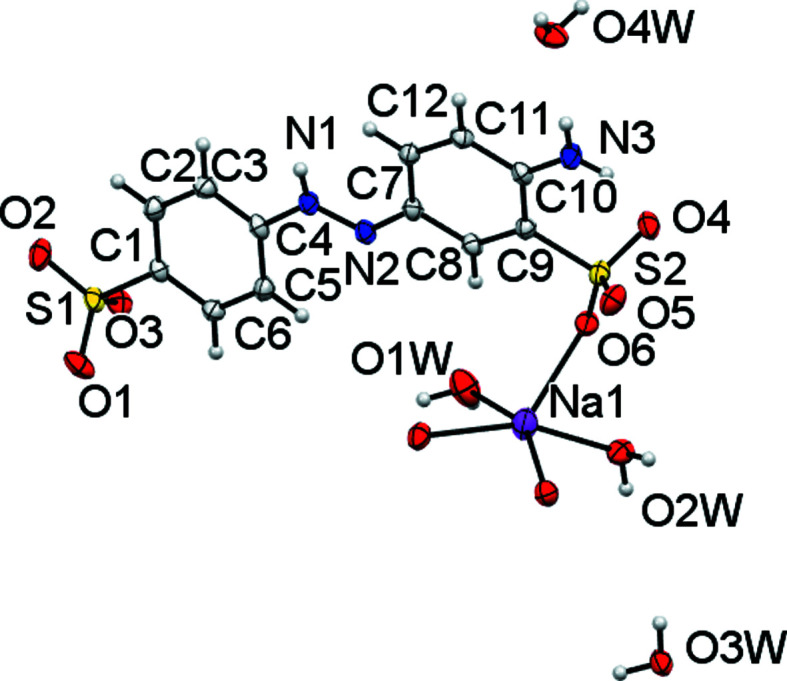
The asymmetric unit of (III) expanded to show the coordination shell about Na1. The minor-disorder com­ponent at O4*W* is not shown. Non-H atoms are shown as 50% probability displacement ellipsoids and H atoms are drawn as small spheres of arbitrary size.

**Figure 7:**
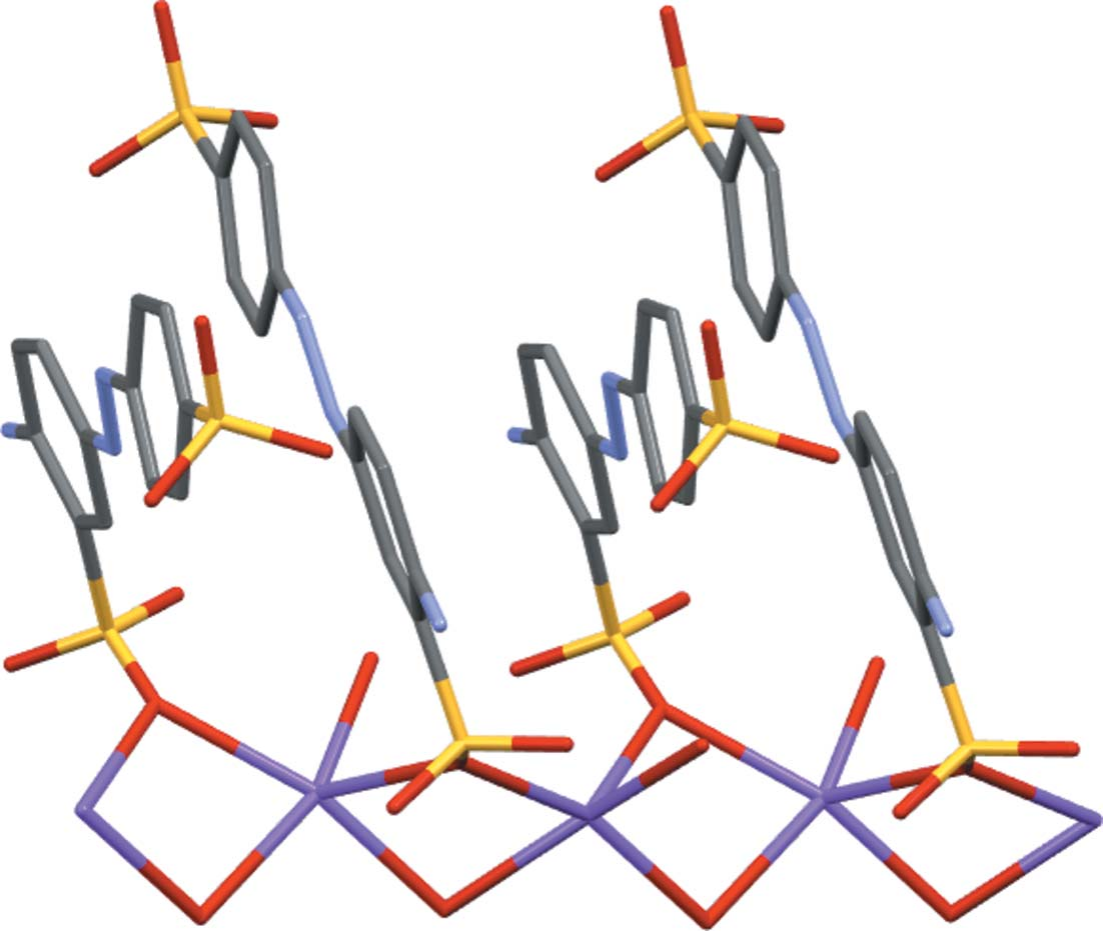
Part of the one-dimensional coordination polymer of (III).

**Figure 8:**
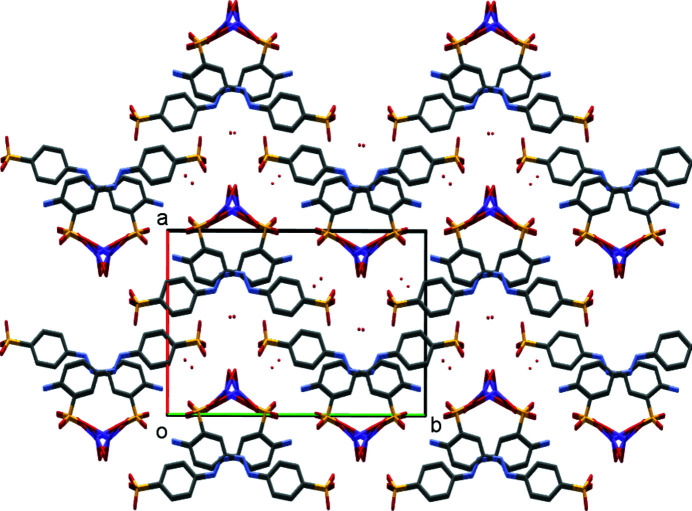
Packing diagram of (III), viewed down the *c* axis. H atoms have been omitted for clarity. Note the hydrate channels that extend parallel to the *c* axis.

Structure (III) was obtained from aqueous recrystallization of the commercial product called ‘Acid Yellow 9, monosodium salt’. An inter­esting problem here was to discover the protonation site. The crystal structures of three acidic sulfonated azo­benzene-based dyes with amino substituents are known. 4-Amino­azo­benzene-4′-sulfonic acid crystallizes with proton­ation of the amino group, giving an –NH_3_-bearing zwitterion, whilst the other two known structures crystallize with protonation of the azo N atom furthest from the neutral –NH_2_ group (Lu *et al.*, 2009 ▸; Miyano *et al.*, 2016 ▸; Kennedy *et al.*, 2020 ▸). The azo group is the commonest protonation site for the free acid forms of sulfonated azo dyes that do not bear a more basic substituent (Kennedy *et al.*, 2001 ▸, 2020 ▸). The asymmetric unit of (III) was found to contain an Na centre, a monoanionic *L*2 ligand with protonation at azo atom N1, two metal-coordinated water ligands and two non-bound water mol­ecules, one of which is disordered (Fig. 6 ▸). Unusually for an Na salt of an aryl sulfonate, only one of the six independent SO_3_ O atoms is involved in bonding to Na. This Na1—O6 inter­action involves the SO_3_ group *meta* to the azo bond. Na1 exists in a distorted square-pyramidal and hence five-coordinate environment, where one bond is to a terminal water ligand and the other four bonds (from two water ligands and two SO_3_ groups) all bridge to neighbouring Na centres (see Table 6 ▸ for geometric details). The Na—O bond lengths of (III) [range 2.275 (2)–2.425 (2) Å] are understandably shorter than those of the six-coordinate Na centres of (I). An inter­esting detail is that in (III) the Na-to-OH_2_ distances are shorter that the Na-to-SO_3_ distances. This is the opposite of the case in (I). The one-dimensional coordination polymers in (III) are formed by chains of [Na1—O2*W*—Na1—O6] rings and propagate parallel to the crystallographic *c* direction. Each chain is asymmetric, with the *L*2 anions on one side and the water ligands on the other (Fig. 7 ▸). This structure is thus unlike those of the mono­sulfonated azo Na salts as, despite having an extra potential metal-bonding group in the form of the second SO_3_ substituent, it does not form a higher-dimensional coordination polymer. A further difference is highlighted by Fig. 8 ▸, which shows that (III) is not a simple alternating layer structure. Note the hydrate channels running parallel to *c*. A reason for this may be that the simple alternate layering seen elsewhere is a function of the azo anions’ approximation to linear spacers, with hydro­philic head and tail groups separated by a hydro­phobic central region (Kennedy *et al.*, 2009 ▸). As *L*2 is protonated on the azo group, this introduces a hydro­philic group and strong hydrogen-bond donor to the centre of the azo anion. It may be that the need to provide a hydrogen-bond acceptor to this formally charged N—H group is what breaks the otherwise common simple layering motif (Table 7 ▸). In this respect, the packing of (III) is more similar to the packing of free acid sulfonated azo structures than it is to the packing of equivalent salt forms (Kennedy *et al.*, 2020 ▸).

**Figure 9:**
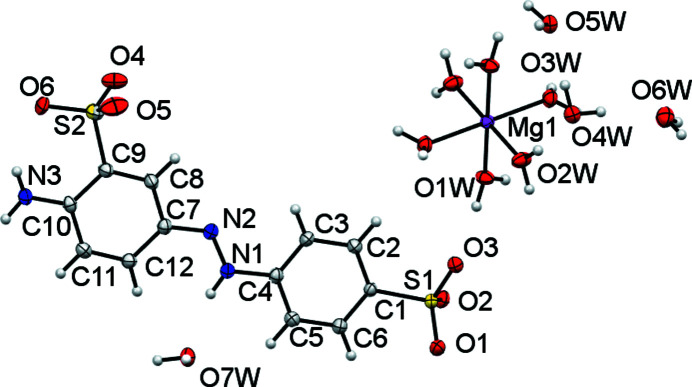
The asymmetric unit of (IV) expanded to show the coordination shell about Mg1. The minor-disorder com­ponents of the sulfonate groups of S2 and the O7*W* water mol­ecule are not shown. Non-H atoms are shown as 50% probability displacement ellipsoids and H atoms are drawn as small spheres of arbitrary size.

**Figure 10:**
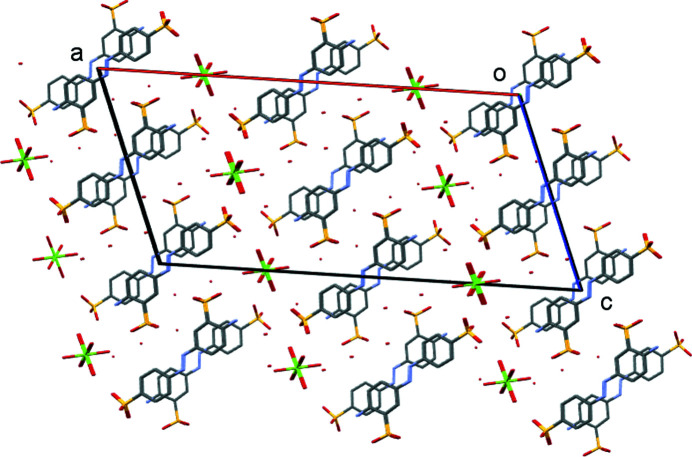
Packing diagram of (IV), viewed down the *b* axis. H atoms have been omitted for clarity. Note the solvent water mol­ecules lying within the layers of azo dianions that lie parallel to the *bc* plane.

All known Mg salt forms of sulfonated azo dyes and pigments are solvent-separated ion pairs, with no direct bond between Mg and SO_3_ (Kennedy *et al.*, 2006 ▸, 2009 ▸, 2012 ▸). As is shown in Fig. 9 ▸, the structure of (IV) is also of this type. Its asymmetric unit contains an *L*2 anion that is protonated at the azo N1 atom, half of an octa­hedral [Mg(OH_2_)]_6_ dication (with Mg1 situated at a crystallographic inversion centre) and four noncoordinated water mol­ecules (Table 8 ▸). One of the water mol­ecules and the SO_3_ group *ortho* to NH_2_ are disordered. As shown in the packing diagram (Fig. 10 ▸), there are hydro­philic layers that extend parallel to the *bc* plane. The organic anions lie between these but their azo­benzene cores do not form continuous hydro­phobic layers – instead water mol­ecules are dispersed within these layers. Thus, rather than true two-dimensional layers, the hydro­phobic azo­benzene units form stacks parallel to the *b* direction surrounded by [Mg(OH_2_)_6_]^2+^ ions and water mol­ecules. As with (III) above, the protonation of the azo unit at the centre of the anion appears to mitigate against the simple alternating layer structures seen elsewhere. In both (III) and (IV), the protonated azo group acts as a hydrogen-bond donor to water mol­ecules (see Tables 7 ▸ and 9 ▸).

**Figure 11:**
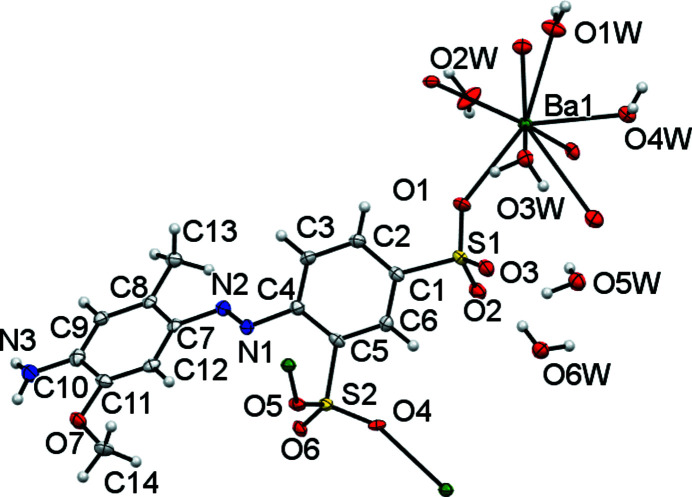
The asymmetric unit of (V) expanded to show the coordination shell about Ba1 and all dative bonds originating from the modelled dianion. Non-H atoms are shown as 50% probability displacement ellipsoids and H atoms are drawn as small spheres of arbitrary size.

Fig. 11 ▸ shows the contents of the asymmetric unit of (V) extended to give the com­plete coordination geometry (Table 10 ▸). The asymmetric unit consists of an azo dianion, a Ba^II^ cation with four coordinated water ligands and two non-bound water mol­ecules. The Ba centre is nona­coordinated, with three bonds to O atoms of SO_3_ groups and six bonds to water ligands. The Ba—O—Ba bridges all involve water O atoms. Both SO_3_ groups inter­act with the Ba atom, with the group *ortho* to the azo group making two Ba—O bonds and the *para* SO_3_ group making one bond. This is notable as *ortho* SO_3_ groups are generally unfavourable coordination sites com­pared to *para* SO_3_ groups (Kennedy *et al.*, 2009 ▸). As with both *L*2 structures, here the amino group of *L*3 takes no part in coordination to the metal atom.

**Figure 12:**
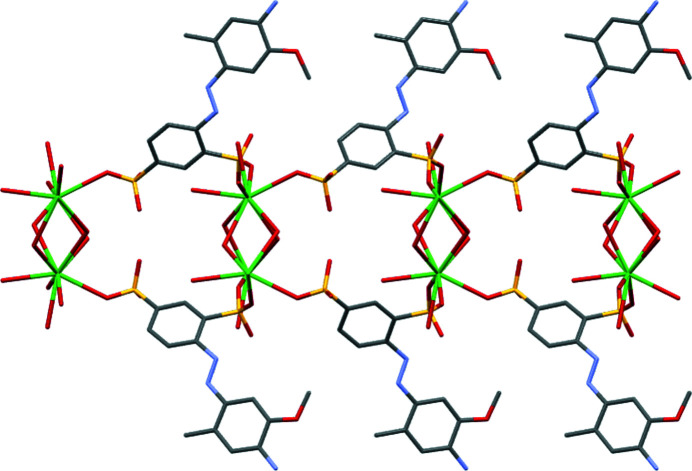
Part of the two-dimensional coordination polymer of (V), viewed down the *a* axis, showing the coordination polymer extending by SO_3_ coordination parallel to the *b* direction.

**Figure 13:**
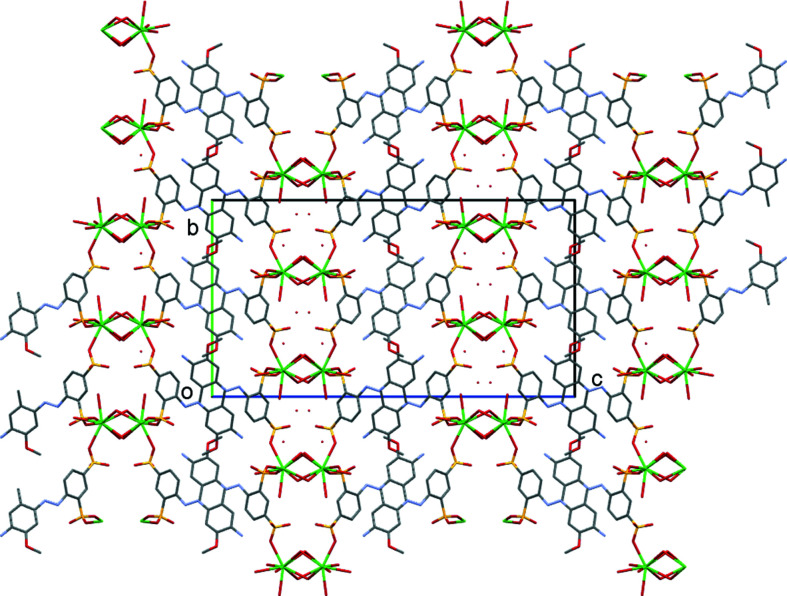
Packing diagram of (V), viewed down the *a* axis. H atoms have been omitted for clarity.

Complex (V) forms a two-dimensional coordination polymer. Ba—O—Ba bridges involving the water mol­ecules extend the polymer parallel to the *a* direction, whilst parallel to the *b* direction, the polymer propagates through the coordination of the two SO_3_ groups to give the large [Ba(OH_2_)_4_Ba(*L*3)]_2_ cyclic structures shown in Fig. 12 ▸. The overall packing (Fig. 13 ▸) shows a layered structure with hydro­phobic and hydro­philic layers parallel to the *ab* plane. As with (III) and (IV), the amine group of (V) is essentially planar rather than pyramidal. However, it differs by acting as a hydrogen-bond donor to only SO_3_ groups (Table 11 ▸), whilst the amine groups of (III) and (IV) donate hydrogen bonds to both SO_3_ and water groups. None of the amine groups act as hydrogen-bond acceptors. Azo atom N1 of (V) does act as a hydrogen-bond acceptor from water, as do both azo N atoms of (I), but this is not the case for any of the other azo N atoms, see hydrogen-bond tables for details.

The literature on the Ba salt forms of mono­sulfonated azo dyes predicts structures with no bridging water ligands and with discrete coordination com­plexes or simple one-dimensional coordination polymers (Kennedy *et al.*, 2004 ▸, 2009 ▸). Neither prediction is true for di­sulfonate (V).

For *L*2, with its protonated azo group, the N=N bond lengths of (III) and (IV) are 1.294 (3) and 1.294 (4) Å, respectively. The N2—C7 bond lengths are also equivalent at 1.341 (3) and 1.342 (4) Å. These values are as expected for a protonated azo unit bound to an aniline fragment and, despite being for an anionic ligand, are close matches to those found for the overall neutral but zwitterionic free acid forms of those mono­sulfonated azo dyes which also feature protonated azo groups (Kennedy *et al.*, 2020 ▸). At 1.256 (3) and 1.432 (2) Å, the N=N and N2—C7 bond lengths of *L*1 in (II) are clearly much shorter and longer, respectively, than their equivalents in *L*2. They fit well with the ranges found for the 4,4′ isomer and with those found for mono­sulfonated azo species with no strong electron-donating ring substituents (Soegiarto *et al.*, 2009 ▸, 2010 ▸, 2011 ▸; Kennedy *et al.*, 2001 ▸, 2020 ▸). The N=N bond in (I) is 1.262 (4) Å and is thus outside the ranges of the literature structures above; however, the difference is not statistically significant. For (V), the N=N and N2—C7 bond lengths of *L*3 are inter­mediate between the lengths reported for *L*1 and *L*2 above at 1.277 (6) and 1.393 (6) Å. Such distortions from the expected geometry of azo­benzene (Harada & Ogawa, 2004 ▸) can be explained by the resonance electron-donating ability of the NH_2_ group *para* to the azo group (Kennedy *et al.*, 2020 ▸). The values found for dianion *L*3 are, however, slightly more distorted from the azo­benzene base than has been found for metal com­plexes of related monoanions, such as 4-amino­azo­benzene-4′-sulfonate (Kennedy *et al.*, 2004 ▸; Lu *et al.*, 2009 ▸). A final point about the geometries of the azo species herein is that in (I)–(IV), the azo moiety is essentially planar [range of dihedral angles between ring planes = 0.00 (6)–14.13 (6)°]. In com­parison, the dianion of (V) is distinctly twisted [dihedral angle between the ring planes = 34.0 (2)°] and stepped [*e.g.* atom N2 lies 0.905 (9) Å out of the plane defined by atoms C1–C6].

## Conclusion   

Compounds (I) and (II) both contain the simple di­sulfonate *L*1 and both have structures that fit with the structural types seen for equivalent mono­sulfonate salt species – they give the expected dimensionality coordination polymers in which the bonding roles of water ligands are predictable and their packing structures have the expected alternating layer motifs (Kennedy *et al.*, 2004 ▸). However, the other three structures presented herein do not have the same structural features as their mono­sulfonate cognates. Structures (III) and (IV) both contain the monoanion *L*2. Neither adopts the expected simple alternating layer structure and Na salt (III) is a one-dimensional coordination polymer rather than the expected two- or three-dimensional coordination polymer. The strong hydrogen-bonding N—H group at the centre of *L*2 is a feature not seen in other salt structures. This difference gives a rational explanation for the difference in packing behaviour. Finally, the Ba salt of *L*3, *i.e.* (V), does give the expected layered packing, but has metal-centre-bridging water ligands and an unexpected two-dimensional rather than a one-dimensional coordination polymer structure. The extra dimensionality of the coordination polymer may simply be related to the extra SO_3_ group in *L*3 com­pared to literature structures, but it is less clear why the coordination role of the water ligands should also change.

## Supplementary Material

Crystal structure: contains datablock(s) I, II, III, IV, V, global. DOI: 10.1107/S2053229620012735/yf3208sup1.cif

Structure factors: contains datablock(s) I. DOI: 10.1107/S2053229620012735/yf3208Isup2.hkl

Structure factors: contains datablock(s) II. DOI: 10.1107/S2053229620012735/yf3208IIsup3.hkl

Structure factors: contains datablock(s) III. DOI: 10.1107/S2053229620012735/yf3208IIIsup4.hkl

Structure factors: contains datablock(s) IV. DOI: 10.1107/S2053229620012735/yf3208IVsup5.hkl

Structure factors: contains datablock(s) V. DOI: 10.1107/S2053229620012735/yf3208Vsup6.hkl

CCDC references: 2032738, 2032737, 2032736, 2032735, 2032734

## Figures and Tables

**Table:** 

	(I)	(II)	(III)
Crystal data			
Chemical formula	[Na_2_(C_12_H_8_N_2_O_6_S_2_)(H_2_O)_4_]	[Ca(C_12_H_8_N_2_O_6_S_2_)(H_2_O)_4_]	[Na(C_12_H_10_N_3_O_6_S_2_)(H_2_O)_2_]·2H_2_O
*M*_r_	458.37	452.47	451.40
Crystal system, space group	Monoclinic, *C*2/*c*	Triclinic, *P* 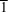	Monoclinic, *P*2_1_/*c*
Temperature (K)	130	123	150
*a*, *b*, *c* (Å)	21.2141 (9), 5.5370 (3), 15.3045 (8)	6.3875 (2), 6.7470 (2), 11.3030 (5)	13.9454 (18), 19.517 (3), 6.9014 (9)
α, β, γ (°)	90, 90.310 (2), 90	94.289 (2), 103.160 (2), 108.456 (2)	90, 93.838 (2), 90
*V* (Å^3^)	1797.68 (16)	444.21 (3)	1874.2 (4)
*Z*	4	1	4
Radiation type	Mo *K*α	Mo *K*α	Synchrotron, λ = 0.6775 Å
μ (mm^−1^)	0.40	0.65	0.32
Crystal size (mm)	0.50 × 0.32 × 0.08	0.50 × 0.25 × 0.05	0.50 × 0.01 × 0.01

Data collection			
Diffractometer	Nonius KappaCCD	Nonius Kappa CCD	Bruker APEXII
Absorption correction	–	–	Multi-scan (*SADABS*; Bruker, 2012 ▸)
*T*_min_, *T*_max_	–	–	0.676, 1.000
No. of measured, independent and observed [*I* > 2σ(*I*)] reflections	3500, 1865, 1414	3837, 2038, 1775	15360, 3531, 2772
*R*_int_	0.035	0.020	0.049
(sin θ/λ)_max_ (Å^−1^)	0.629	0.651	0.608

Refinement			
*R*[*F* ^2^ > 2σ(*F* ^2^)], *wR*(*F* ^2^), *S*	0.038, 0.090, 1.04	0.027, 0.070, 1.05	0.040, 0.107, 1.04
No. of reflections	1865	2038	3531
No. of parameters	145	140	311
No. of restraints	6	6	15
H-atom treatment	H atoms treated by a mixture of independent and constrained refinement	H atoms treated by a mixture of independent and constrained refinement	H atoms treated by a mixture of independent and constrained refinement
Δρ_max_, Δρ_min_ (e Å^−3^)	0.43, −0.32	0.40, −0.46	0.35, −0.44

**Table:** 

	(IV)	(V)
Crystal data		
Chemical formula	[Mg(H_2_O)_6_](C_12_H_10_N_3_O_6_S_2_)_2_·8H_2_O	[Ba(C_14_H_13_N_3_O_7_S_2_)(H_2_O)_4_]·2H_2_O
*M*_r_	989.23	644.83
Crystal system, space group	Monoclinic, *C*2/*c*	Orthorhombic, *P* *b* *c* *a*
Temperature (K)	123	123
*a*, *b*, *c* (Å)	36.896 (3), 6.7806 (4), 17.9140 (12)	7.1293 (4), 18.8368 (11), 34.752 (2)
α, β, γ (°)	90, 111.178 (9), 90	90, 90, 90
*V* (Å^3^)	4179.0 (6)	4667.0 (5)
*Z*	4	8
Radiation type	Cu *K*α	Mo *K*α
μ (mm^−1^)	3.12	1.95
Crystal size (mm)	0.5 × 0.05 × 0.03	0.25 × 0.10 × 0.04

Data collection		
Diffractometer	Oxford Diffraction Gemini S	Nonius KappaCCD
Absorption correction	Multi-scan (*CrysAlis PRO*; Rigaku OD, 2019 ▸)	Multi-scan (*SADABS*; Bruker, 2012 ▸)
*T*_min_, *T*_max_	0.572, 1.000	0.448, 0.743
No. of measured, independent and observed [*I* > 2σ(*I*)] reflections	7541, 4093, 3287	7914, 4489, 3554
*R*_int_	0.039	0.037
(sin θ/λ)_max_ (Å^−1^)	0.621	0.616

Refinement		
*R*[*F* ^2^ > 2σ(*F* ^2^)], *wR*(*F* ^2^), *S*	0.050, 0.143, 1.06	0.042, 0.096, 1.15
No. of reflections	4093	4489
No. of parameters	359	344
No. of restraints	110	20
H-atom treatment	H atoms treated by a mixture of independent and constrained refinement	H atoms treated by a mixture of independent and constrained refinement
Δρ_max_, Δρ_min_ (e Å^−3^)	0.80, −0.38	1.65, −1.23

Computer programs: *COLLECT* (Hooft, 1998 ▸), *SAINT* (Bruker, 2012 ▸), *CrysAlis PRO* (Rigaku OD, 2019 ▸), *DENZO* (Otwinowski & Minor, 1997 ▸), *SHELXS* (Sheldrick, 2015 ▸), *SIR92* (Altomare *et al.*, 1994 ▸), *WinGX* (Farrugia, 2012 ▸), *Mercury* (Macrae *et al.*, 2020 ▸), *ORTEP-3* (Farrugia, 2012 ▸) and *SHELXL2014* (Sheldrick, 2015 ▸).

**Table 2:** Selected geometric parameters (Å, °) for (I)

Na1—O3^i^	2.4174 (19)	Na2—O2	2.3340 (18)
Na1—O3^ii^	2.4175 (19)	Na2—O1*W*	2.3688 (17)
Na1—O1	2.419 (2)	Na2—O1*W* ^iv^	2.3688 (17)
Na1—O1^iii^	2.419 (2)	Na2—O2*W* ^iv^	2.4480 (17)
Na1—O1*W* ^iii^	2.5019 (18)	Na2—O2*W*	2.4480 (17)
Na1—O1*W*	2.5019 (18)	N1—N1^v^	1.262 (4)
Na2—O2^iv^	2.3340 (18)	N1—C3	1.431 (3)
			
O3^i^—Na1—O3^ii^	100.81 (10)	O2^iv^—Na2—O2	180.0
O3^i^—Na1—O1	85.49 (6)	O2^iv^—Na2—O1*W*	91.49 (7)
O3^ii^—Na1—O1	163.62 (6)	O2—Na2—O1*W*	88.51 (7)
O1—Na1—O1^iii^	92.59 (9)	O1*W*—Na2—O1*W* ^iv^	180.0
O3^i^—Na1—O1*W* ^iii^	86.52 (7)	O2—Na2—O2*W* ^iv^	98.30 (6)
O3^ii^—Na1—O1*W* ^iii^	75.28 (6)	O1*W*—Na2—O2*W* ^iv^	87.42 (6)
O1—Na1—O1*W* ^iii^	90.17 (6)	O2—Na2—O2*W*	81.70 (6)
O1—Na1—O1*W*	109.77 (6)	O1*W*—Na2—O2*W*	92.58 (6)
O1*W* ^iii^—Na1—O1*W*	151.40 (10)		

Symmetry codes: (i) 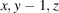; (ii) 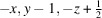; (iii) 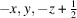; (iv) 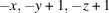; (v) 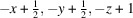.

**Table 3:** Hydrogen-bond geometry (Å, °) for (I)

*D*—H⋯*A*	*D*—H	H⋯*A*	*D*⋯*A*	*D*—H⋯*A*
O1*W*—H2*W*⋯O2*W* ^i^	0.87 (1)	2.07 (2)	2.919 (3)	163 (3)
O1*W*—H1*W*⋯O2^i^	0.87 (1)	2.29 (2)	3.044 (3)	145 (3)
O1*W*—H1*W*⋯O3^i^	0.87 (1)	2.32 (3)	3.005 (3)	136 (3)
O2*W*—H3*W*⋯O1^iii^	0.87 (1)	1.94 (1)	2.807 (2)	175 (3)
O2*W*—H4*W*⋯N1^vi^	0.87 (1)	2.22 (1)	3.076 (3)	168 (3)

Symmetry codes: (i) 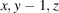; (iii) 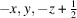; (vi) 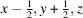.

**Table 4:** Selected geometric parameters (Å, °) for (II)

Ca1—O3	2.3050 (11)	Ca1—O2*W* ^i^	2.3385 (12)
Ca1—O3^i^	2.3051 (11)	Ca1—O2*W*	2.3385 (12)
Ca1—O1*W*	2.3235 (12)	N1—N1^ii^	1.256 (3)
Ca1—O1*W* ^i^	2.3236 (12)	N1—C3	1.432 (2)
			
O3—Ca1—O3^i^	180.0	O1*W*—Ca1—O2*W* ^i^	90.52 (5)
O3—Ca1—O1*W*	87.66 (4)	O3—Ca1—O2*W*	86.93 (5)
O3—Ca1—O1*W* ^i^	92.34 (4)	O1*W*—Ca1—O2*W*	89.48 (5)
O1*W*—Ca1—O1*W* ^i^	180.00 (6)	O2*W* ^i^—Ca1—O2*W*	180.0
O3—Ca1—O2*W* ^i^	93.07 (4)		

Symmetry codes: (i) 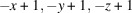; (ii) 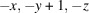.

**Table 5:** Hydrogen-bond geometry (Å, °) for (II)

*D*—H⋯*A*	*D*—H	H⋯*A*	*D*⋯*A*	*D*—H⋯*A*
O1*W*—H1*W*⋯O2^iii^	0.87 (1)	2.01 (1)	2.8521 (17)	162 (2)
O1*W*—H2*W*⋯O1^iv^	0.86 (1)	2.00 (1)	2.8454 (17)	165 (2)
O2*W*—H3*W*⋯O2^iv^	0.86 (1)	1.95 (1)	2.8119 (16)	174 (2)
O2*W*—H4*W*⋯O1^v^	0.86 (1)	1.94 (1)	2.7907 (16)	168 (2)

Symmetry codes: (iii) 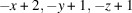; (iv) 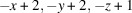; (v) 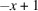, 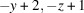.

**Table 6:** Selected geometric parameters (Å, °) for (III)

Na1—O1*W*	2.275 (2)	N1—N2	1.294 (3)
Na1—O2*W* ^i^	2.335 (2)	N1—C4	1.411 (3)
Na1—O2*W*	2.369 (2)	N2—C7	1.341 (3)
Na1—O6	2.409 (2)	N3—C10	1.316 (3)
Na1—O6^i^	2.425 (2)		
			
O1*W*—Na1—O2*W* ^i^	154.18 (9)	O2*W*—Na1—O6	75.46 (7)
O1*W*—Na1—O2*W*	96.67 (9)	O1*W*—Na1—O6^i^	85.06 (8)
O2*W* ^i^—Na1—O2*W*	95.44 (7)	O2*W* ^i^—Na1—O6^i^	75.79 (7)
O1*W*—Na1—O6	88.23 (8)	O2*W*—Na1—O6^i^	159.34 (8)
O2*W* ^i^—Na1—O6	116.93 (8)	O6—Na1—O6^i^	125.20 (8)

Symmetry code: (i) 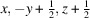.

**Table 7:** Hydrogen-bond geometry (Å, °) for (III)

*D*—H⋯*A*	*D*—H	H⋯*A*	*D*⋯*A*	*D*—H⋯*A*
N1—H1N⋯O3*W* ^ii^	0.84 (3)	2.10 (3)	2.878 (3)	153 (3)
N3—H2N⋯O4*W*	0.82 (3)	2.04 (3)	2.847 (5)	170 (3)
N3—H2N⋯O5*W*	0.82 (3)	2.05 (4)	2.843 (11)	163 (3)
N3—H3N⋯O4	0.81 (3)	2.60 (3)	3.084 (3)	120 (3)
N3—H3N⋯O4^iii^	0.81 (3)	2.20 (3)	2.923 (3)	150 (3)
N3—H3N⋯O5	0.81 (3)	2.61 (3)	3.095 (3)	120 (3)
O1*W*—H1*W*⋯O5^iv^	0.88 (1)	1.93 (2)	2.773 (3)	160 (4)
O1*W*—H2*W*⋯O4^i^	0.88 (1)	1.93 (2)	2.756 (3)	157 (4)
O2*W*—H3*W*⋯O3^v^	0.88 (1)	1.90 (1)	2.774 (2)	176 (4)
O2*W*—H4*W*⋯O1^vi^	0.88 (1)	1.88 (1)	2.746 (3)	171 (3)
O3*W*—H5*W*⋯O2^vii^	0.86 (1)	2.03 (1)	2.866 (3)	164 (3)
O3*W*—H6*W*⋯O3^vi^	0.87 (1)	2.22 (1)	3.081 (3)	171 (3)
O4*W*—H7*W*⋯O3^i^	0.88 (1)	2.02 (1)	2.887 (5)	175 (4)
O4*W*—H8*W*⋯O1^iv^	0.88 (1)	1.97 (1)	2.846 (5)	173 (5)
O5*W*—H9*W*⋯O3^i^	0.88 (1)	2.08 (2)	2.947 (13)	170 (10)
O5*W*—H10*W*⋯O1^iv^	0.88 (1)	1.89 (2)	2.765 (9)	171 (10)

Symmetry codes: (i) 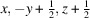; (ii) 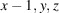; (iii) 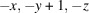; (iv) 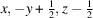; (v) 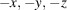; (vi) 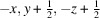; (vii) 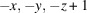.

**Table 8:** Selected geometric parameters (Å, °) for (IV)

Mg1—O2*W*	2.0322 (19)	N1—C4	1.413 (3)
Mg1—O1*W*	2.0472 (18)	N2—C7	1.342 (4)
Mg1—O3*W*	2.0769 (19)	N3—C10	1.309 (4)
N1—N2	1.294 (4)		
			
O2*W* ^i^—Mg1—O2*W*	180.0	O1*W*—Mg1—O3*W* ^i^	88.07 (8)
O2*W* ^i^—Mg1—O1*W*	88.72 (8)	O2*W*—Mg1—O3*W*	91.69 (8)
O2*W*—Mg1—O1*W*	91.28 (8)	O1*W*—Mg1—O3*W*	91.93 (8)
O1*W*—Mg1—O1*W* ^i^	180.0	O3*W* ^i^—Mg1—O3*W*	180.0
O2*W*—Mg1—O3*W* ^i^	88.31 (8)		

Symmetry code: (i) 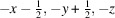.

**Table 9:** Hydrogen-bond geometry (Å, °) for (IV)

*D*—H⋯*A*	*D*—H	H⋯*A*	*D*⋯*A*	*D*—H⋯*A*
N1—H1N⋯O7*W*	0.84 (4)	1.99 (5)	2.80 (2)	164 (4)
N1—H1N⋯O8*W*	0.84 (4)	2.00 (6)	2.83 (4)	170 (4)
N3—H2N⋯O5*W* ^ii^	0.87 (4)	2.02 (4)	2.881 (3)	174 (4)
N3—H3N⋯O6	0.84 (5)	2.03 (5)	2.700 (7)	137 (4)
N3—H3N⋯O6*A*	0.84 (5)	2.10 (5)	2.763 (14)	136 (4)
O1*W*—H1*W*⋯O3	0.87 (1)	1.92 (1)	2.769 (3)	167 (3)
O1*W*—H2*W*⋯O1^iii^	0.87 (1)	1.84 (1)	2.714 (3)	177 (4)
O2*W*—H3*W*⋯O4*W*	0.87 (1)	1.88 (1)	2.727 (3)	165 (3)
O2*W*—H4*W*⋯O2^iv^	0.87 (1)	1.95 (1)	2.812 (3)	173 (4)
O3*W*—H5*W*⋯O4*W* ^v^	0.87 (1)	1.85 (1)	2.707 (3)	169 (4)
O3*W*—H6*W*⋯O6^vi^	0.87 (1)	2.04 (2)	2.897 (6)	169 (4)
O3*W*—H6*W*⋯O6*A* ^vi^	0.87 (1)	2.18 (2)	2.983 (11)	153 (3)
O4*W*—H8*W*⋯O5*W*	0.87 (1)	1.95 (2)	2.803 (3)	166 (4)
O4*W*—H7*W*⋯O6*W*	0.87 (1)	1.86 (1)	2.706 (3)	162 (3)
O5*W*—H10*W*⋯O2^i^	0.87 (1)	2.16 (3)	2.829 (3)	133 (3)
O5*W*—H9*W*⋯O3^vii^	0.87 (1)	1.99 (1)	2.820 (3)	160 (3)
O5*W*—H10*W*⋯O3^viii^	0.87 (1)	2.52 (3)	3.251 (3)	141 (3)
O6*W*—H11*W*⋯O4^vi^	0.87 (1)	2.18 (2)	3.028 (13)	167 (4)
O6*W*—H11*W*⋯O4*A* ^vi^	0.87 (1)	1.94 (3)	2.81 (2)	174 (4)
O6*W*—H12*W*⋯O5^ix^	0.87 (1)	2.02 (2)	2.878 (8)	170 (5)
O6*W*—H12*W*⋯O5*A* ^ix^	0.87 (1)	2.43 (2)	3.276 (14)	163 (4)
O6*W*—H12*W*⋯O6*A* ^ix^	0.87 (1)	2.52 (3)	3.220 (17)	139 (4)
O7*W*—H13*W*⋯O5^x^	0.89 (1)	2.35 (6)	2.89 (2)	119 (5)
O7*W*—H14*W*⋯O6*W* ^ii^	0.88 (1)	2.52 (3)	3.354 (18)	159 (5)
O7*W*—H14*W*⋯O4^xi^	0.88 (1)	2.35 (5)	2.92 (2)	123 (4)
O8*W*—H16*W*⋯O5*A* ^x^	0.89	2.01	2.87 (4)	163
O8*W*—H15*W*⋯O4*A* ^xi^	0.88	2.18	2.81 (4)	128

Symmetry codes: (i) 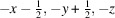; (ii) 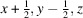; (iii) 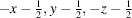; (iv) 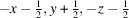; (v) 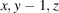; (vi) 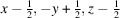; (vii) 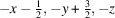; (viii) 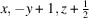; (ix) 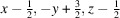; (x) 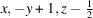; (xi) 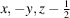.

**Table 10:** Selected geometric parameters (Å, °) for (V)

Ba1—O2*W*	2.704 (4)	Ba1—O3*W*	2.911 (4)
Ba1—O1*W*	2.747 (4)	Ba1—O3*W* ^iii^	3.105 (4)
Ba1—O4^i^	2.753 (4)	N1—N2	1.277 (6)
Ba1—O1	2.759 (4)	N1—C4	1.426 (6)
Ba1—O5^ii^	2.788 (4)	N2—C7	1.393 (6)
Ba1—O4*W*	2.819 (4)	N3—C10	1.354 (7)
			
O2*W*—Ba1—O1*W*	72.69 (14)	O1—Ba1—O3*W*	85.63 (11)
O2*W*—Ba1—O4^i^	68.74 (12)	O5^ii^—Ba1—O3*W*	87.04 (11)
O1*W*—Ba1—O4^i^	83.39 (12)	O4*W*—Ba1—O3*W*	76.65 (11)
O2*W*—Ba1—O1	73.66 (13)	O2*W*—Ba1—O3*W* ^iii^	124.26 (12)
O1*W*—Ba1—O1	146.32 (12)	O1*W*—Ba1—O3*W* ^iii^	126.70 (12)
O4^i^—Ba1—O1	85.75 (11)	O4^i^—Ba1—O3*W* ^iii^	64.01 (10)
O2*W*—Ba1—O5^ii^	63.22 (12)	O1—Ba1—O3*W* ^iii^	75.02 (10)
O1*W*—Ba1—O5^ii^	85.47 (12)	O5^ii^—Ba1—O3*W* ^iii^	147.67 (10)
O4^i^—Ba1—O5^ii^	131.85 (10)	O4*W*—Ba1—O3*W* ^iii^	60.15 (10)
O1—Ba1—O5^ii^	78.35 (11)	O3*W*—Ba1—O3*W* ^iii^	73.06 (5)
O2*W*—Ba1—O4*W*	136.43 (13)	O2*W*—Ba1—O4*W* ^iv^	115.49 (11)
O1*W*—Ba1—O4*W*	74.19 (12)	O1*W*—Ba1—O4*W* ^iv^	67.73 (11)
O4^i^—Ba1—O4*W*	80.10 (11)	O4^i^—Ba1—O4*W* ^iv^	146.32 (10)
O1—Ba1—O4*W*	134.80 (11)	O1—Ba1—O4*W* ^iv^	127.91 (11)
O5^ii^—Ba1—O4*W*	140.16 (11)	O5^ii^—Ba1—O4*W* ^iv^	64.82 (10)
O2*W*—Ba1—O3*W*	146.29 (13)	O4*W*—Ba1—O4*W* ^iv^	75.76 (7)
O1*W*—Ba1—O3*W*	123.10 (12)	O3*W*—Ba1—O4*W* ^iv^	58.23 (10)
O4^i^—Ba1—O3*W*	136.99 (11)	O3*W* ^iii^—Ba1—O4*W* ^iv^	120.18 (10)

Symmetry codes: (i) 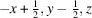; (ii) 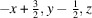; (iii) 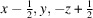; (iv) 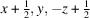.

**Table 11:** Hydrogen-bond geometry (Å, °) for (V)

*D*—H⋯*A*	*D*—H	H⋯*A*	*D*⋯*A*	*D*—H⋯*A*
N3—H1N⋯O2^v^	0.88 (1)	2.27 (2)	3.144 (6)	172 (6)
N3—H2N⋯O6^vi^	0.88 (1)	2.28 (5)	2.984 (6)	138 (6)
O1*W*—H1*W*⋯O2^i^	0.87 (1)	2.21 (3)	2.987 (5)	148 (5)
O1*W*—H2*W*⋯O6*W* ^vii^	0.87 (1)	1.92 (2)	2.766 (6)	161 (6)
O2*W*—H3*W*⋯O6^i^	0.88 (1)	2.03 (3)	2.772 (6)	141 (5)
O2*W*—H4*W*⋯N1^ii^	0.88 (1)	2.10 (2)	2.948 (6)	162 (5)
O3*W*—H5*W*⋯O5*W* ^viii^	0.88 (1)	2.04 (3)	2.805 (5)	145 (4)
O3*W*—H6*W*⋯O5*W* ^iv^	0.88 (1)	1.97 (1)	2.833 (6)	168 (5)
O4*W*—H7*W*⋯O4^vii^	0.88 (1)	2.06 (2)	2.901 (5)	160 (4)
O4*W*—H8*W*⋯O6*W* ^i^	0.88 (1)	1.87 (2)	2.741 (5)	171 (5)
O5*W*—H9*W*⋯O2	0.88 (1)	1.97 (3)	2.805 (5)	158 (6)
O5*W*—H10*W*⋯O5^i^	0.88 (1)	2.17 (3)	2.917 (5)	143 (5)
O6*W*—H11*W*⋯O3	0.88 (1)	1.88 (2)	2.737 (5)	166 (6)
O6*W*—H12*W*⋯O3^iii^	0.88 (1)	1.93 (1)	2.800 (6)	174 (5)

Symmetry codes: (i) 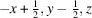; (ii) 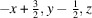; (iii) 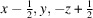; (iv) 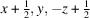; (v) 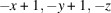; (vi) 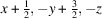; (vii) 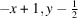, 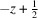; (viii) 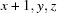.
